# Assessment of Nerve Conduction in Patients With Lower Motor Neuron Facial Paralysis

**DOI:** 10.7759/cureus.35422

**Published:** 2023-02-24

**Authors:** Poornachitra P, Arvind Muthukrishnan

**Affiliations:** 1 Oral Medicine and Radiology, Saveetha Dental College and Hospital, Chennai, IND

**Keywords:** facial palsies, peripheral facial paralysis, lower motor neuron facial palsy, facial paresis, hemifacial paralysis, facial paralysis, facial palsy, neural conduction, nerve conduction, bell palsy

## Abstract

Introduction

Bell’s palsy (BP), a lower motor neuron facial paralysis, commonly causes dysfunction of muscles of facial expression. Nerve conduction electrodiagnostic studies differentiate early-stage minor conduction blocks from later-stage Wallerian degeneration. Nerve conduction studies (NCSs) assess facial nerve function by delivering supramaximal electrical stimulus at the stylomastoid foramen. The amplitude loss percentage of the affected side is calculated with reference to the normal side.

Aim

The study’s aim was to characterize the ncs in BP cases and to evaluate the correlation between the Compound Muscle Action Potential (CMAP) of the muscles affected.

Materials and methods

One hundred and four NCS data of BP cases were retrospectively collected over the period of two years. Statistical analyses of variables were done using the Chi-square test, one-way ANOVA, and Pearson correlation coefficient.

Result

The greater amplitude loss was seen in the orbicularis oris muscle innervated by buccal and mandibular branches of the facial nerve. The bivariate correlation between the Right Nasalis versus Right Orbicularis Oculi and Left Orbicularis Oculi versus Left Nasalis showed a highly significant moderately Strong Positive Correlation with an R-value of 0.687 and 0.558, respectively. The amplitude drop percentage was statistically significant in the affected left and right sides with P values of 0.008 and 0.007 respectively (P value < 0.05). The amplitude drop between the nasalis, orbicularis oculi and orbicularis oris muscles of both sides was statistically significant with a P value of 0.001.

Conclusion

NCS should be mandatorily included as an assessment protocol in BP cases for quantification of nerve degeneration and as a prognostic tool during the course of treatment.

## Introduction

Lower motor neuron facial nerve paralysis also called Bell’s palsy (BP) causes the dysfunction of muscles of facial expression thereby leading to physical, psychological, and functional disabilities [1]. Sir Charles Bell was the first to describe facial paralysis resulting from seventh cranial nerve involvement which he referred to as the respiratory nerve in his case report description [2]. This facial paralysis can involve persons from 10 to 70 years with the involvement of both the right and left side [3] and equal gender predilection [4,5]. The pathogenesis is still not wholly fathomed as no readily identifiable etiology exists. But more upcoming evidence is in favor of the reactivation of Herpes Simplex Virus-1 as a causative agent and the subsequent nerve injury due to inflammatory reaction or cell-mediated autoimmune inflammatory response [6]. The classic features of BP are forehead wrinkling, ptosis of the eyebrow, drooping mouth corner, flattening of the nasolabial fold, and incomplete closure of eyelids, pain in the neck.

Evaluating BP’s prognosis is crucial to formulating treatment plans and improving quality of life [7]. The objective evaluation of nerve degeneration is measured using electroneurography (ENoG), which was first described by Erlo Esslen in 1973 [8] and was further popularized by Ugo Fisch in analyzing nerve degeneration amongst individuals with BP. Nerve conduction studies (NCSs) assess facial nerve function by delivering supramaximal electrical stimulus at the stylomastoid foramen. This supramaximal stimulation current level is required for evoking compound muscle action potential (CMAP) as all functioning nerve fibers have to be simultaneously stimulated [9].

The CMAP amplitude measured in millivolts represents the synchronous discharge of muscle motor units conducted due to external electrical stimulation [10]. The hypothesis is that the CMAP generated in affected facial muscle is directly proportional to the number of affected nerve fibers that have lost their motor function. The paralyzed side of the face is compared with the normal side and the amount of degenerated nerve fibers is quantified. This study aimed to characterize the facial nerve conduction in the facial muscles which influence the prognostic assessment in treatment planning. The objective was also to evaluate the correlation between the CMAP of muscles of facial expression in BP.

## Materials and methods

The study was conducted at the Department of Neurology in our university’s medical college and hospital after obtaining institutional ethical clearance (IHEC Ref. No: IHEC/SDC/OMED-2002/22/429) by conforming to the ethical declarations of Helsinki. The NCS data of BP cases were collected retrospectively from the period of June 2020 to June 2022. Clinically diagnosed BP cases (N= 104) were subjected to an NCS after 72 hours (three days) of onset as this is the period during which Wallerian degeneration would have set in. The test was conducted by a neuro-electrophysiologist under the supervision of the clinical neurologist.

The NCS recording was done on BP patients following the standard procedure [11] in which the bipolar silver chloride surface electrodes were placed over Nasalis, Orbicularis oculi, and Orbicularis Oris muscles after thorough skin preparation with alcohol to reduce impedance created by laden dust particles and oil secretions. The stimulation point of the facial nerve was anterior to the ear lobe as at this site maximum CMAP amplitudes can be obtained with minimum stimulation intensity. The stimulation intensity was kept between 40 and 60 milliamperes and adjusted based on the patient’s comfort.

**Figure 1:**
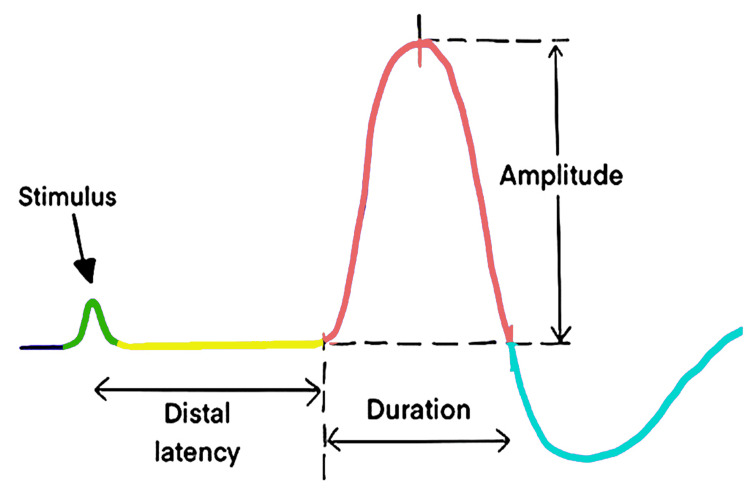
Schematic representation of NCS wave characterization

The recording equipment used was the Allengers Scorpio-4P NCS system (Allengers Medical Systems Ltd) with an NCS sweep speed of 2 milliseconds/Division and an applied voltage of 1 millivolt. For each side, the procedures were repeated twice to verify the results' reproducibility and to select the best CMAP waveform. In an optimal CMAP waveform, the amplitude recorded is measured from the baseline to the negative peak i.e upward deflection (Figure 1). The comparison of CMAP amplitudes between two sides gives an estimate of the severity of axonal degeneration. The facial nerve degeneration severity was calculated using the formula [10], Facial nerve degeneration = (1-n) x 100%, where n = (Amplitude of affected side)/(Amplitude of normal side).

The data were tabulated and formatted in a Microsoft excel sheet, and statistical analyses were done using SPSS software. The association between BP-affected side and amplitude drop percentage was done using the chi-square test and the association between the mean difference of amplitude drop percentages among individual muscles of both sides was measured using one-way ANOVA. The Pearson correlation coefficient was used to assess the linear correlations between affected muscle groups on both sides.

## Results

**Figure 2:**
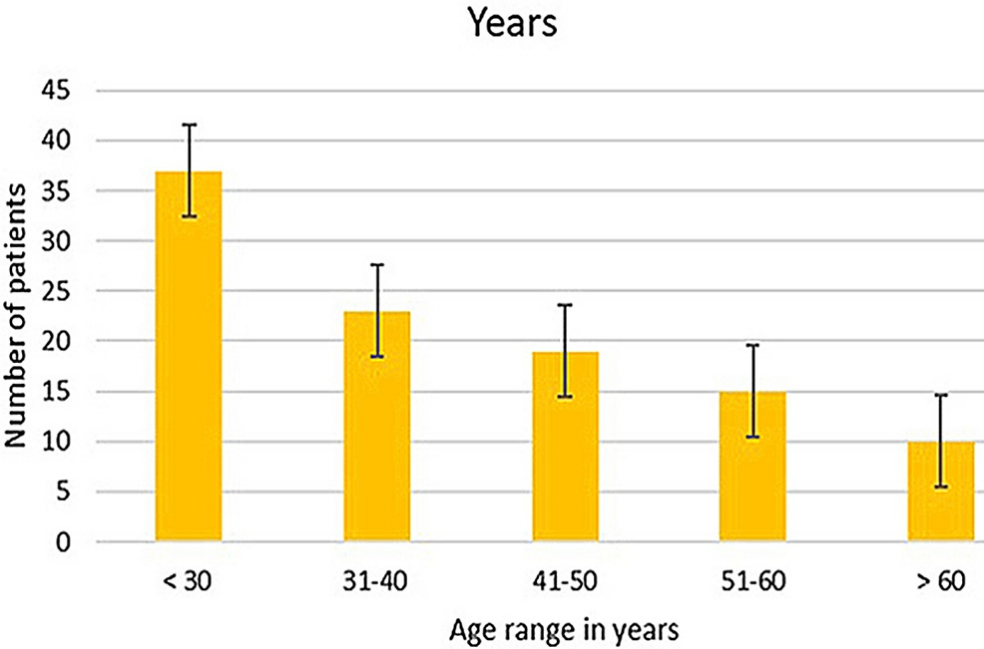
Graphical representation of the distribution of age in years among Bell’s palsy patients. 37 [35.6%) were under the age of 30, 23 [22.1%) were in the 31-40 group, 19 [18.3%) were in the 41-50 group, 15 [14.4%) were in 51-60 and 10 [9.6%) were above 60 years.

The total study participants, N=104 comprised 58 males (55.8%) and 46 females (44.2%) (Table 1). Patients were grouped into five age categories. Out of 104 patients, 37 (35.6%) were under the age group of 30, 23 (22.1%) were 31-40, 19 (18.3%) were 41-50, 15 (14.4%) were 51-60 and 10 (9.6%) were above 60 years (Figure 2). In this study, there was a wide difference in the age of the affected population ranging from a three-year-old child to an 81-year-old adult. Among the affected side, the right side was involved in 51.9% and the left side in 48.1% (Table 2).

**Table 1:** Frequency distribution of gender among Bell’s palsy patients

Gender	Frequency	Percent
Male	58	55.8
Female	46	44.2

**Table 2:** Frequency percentage of the affected side among Bell’s palsy patients

Affected side	Frequency	Percent
Right	54	51.9
Left	50	48.1

**Figure 3:**
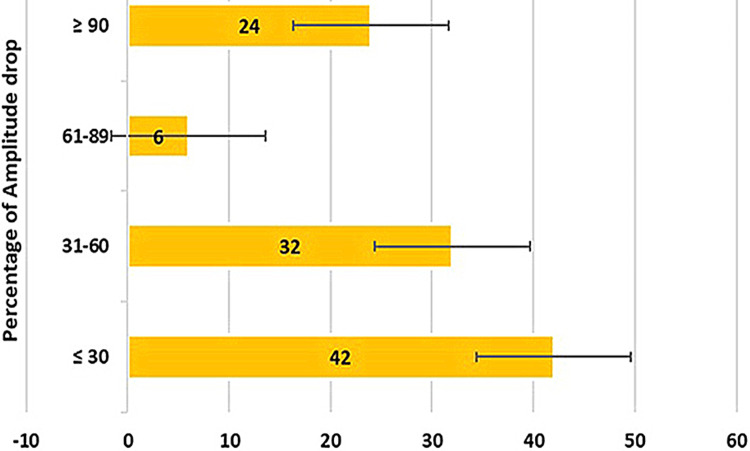
Graphical representation of the frequency distribution of amplitude drop percentage among Bell’s palsy patients.

Figure 3 shows the graphical representation of the frequency distribution of amplitude drop percentage. 40.38% have less than or equal to a 30% amplitude drop, 30.76% have a 31% to 60% drop, 5.76% have a 61% to 89% drop and 23.07% have more than or equal to a 90% amplitude drop. The chi-square test was done to evaluate the association between the BP affected side and amplitude drop percentage (Table 3) and the result was statistically significant in the affected left and right side with P values of 0.008 and 0.007 respectively (P-value < 0.05). A one-way ANOVA test (Table 4) was done to evaluate the mean difference between the variables of different muscle groups on both sides. The amplitude drop between the Nasalis, orbicularis oculi and orbicularis oris muscles of both sides was statistically significant with a P-value of 0.001 (P-value < 0.05). The amplitude loss was higher in the right orbicularis oris followed by the left orbicularis oris.

**Table 3:** Chi-square test showing the association between Bell’s palsy affected side and amplitude drop percentage. P-value < 0.05 was considered statistically significant.

Affected side	Amplitude drop percentage			P-value
	<30	30-60	>60	
Left	28 [56.0%)	11 [22.0%)	11 [22.0%)	0.008*
Right	14 [25.9%)	21 [38.9%)	19 [35.2%)	0.007*”

**Table 4:** One-way Analysis of Variance (ANOVA) result of the mean difference of the variables. P-value <0.05 was considered statistically significant.

Muscle	N	Minimum	Maximum	Mean	Std. Deviation	P-value
Right Nasalis	104	0.01	5.10	1.8746	1.15364	<0.001
Left Nasalis	104	0.09	4.90	1.9798	1.06478	
Right Orbicularis Oculi	104	0.10	4.90	1.8350	1.05482	
Left Orbicularis Oculi	104	0.11	4.10	1.8775	.90611	
Right Orbicularis Oris	104	0.10	285.20	6.3118	27.69495	
Left Orbicularis Oris	104	0.02	18.44	4.0938	2.52111	

**Figure 4:**
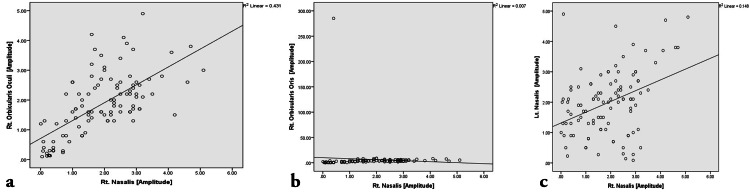
Scatter plot representing the bivariate correlation among affected Nasalis muscle groups on both sides. (a) Correlation between Right Nasalis and Right Orbicularis Oculi. (b) Correlation between Right Nasalis and Right Orbicularis Oris. (c) Correlation between Right Nasalis and Left Nasalis

**Figure 5:**
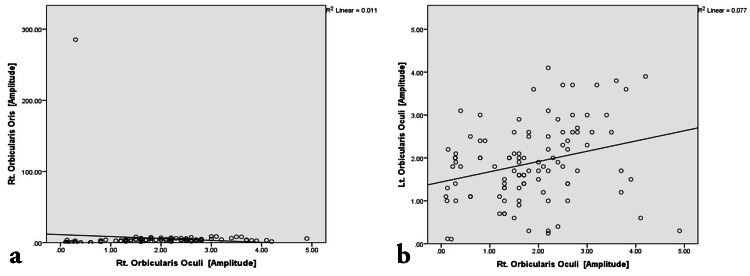
Scatter plot representing the bivariate correlation among affected Orbicularis Oculi muscle groups on both sides. (a) Correlation between Right Orbicularis Oculi and Right Orbicularis Oris. (b) Correlation between Right Orbicularis Oculi and Left Orbicularis Oculi.

**Figure 6:**
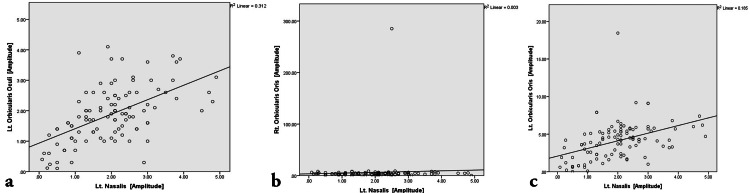
Scatter plot representing the bivariate correlation among affected Left Nasalis muscle groups on both sides. (a) Correlation between Left Nasalis and Left Orbicularis Oculi. (b) Correlation between Left Nasalis and Right Orbicularis Oris. (c) Correlation between Left Nasalis and Left Orbicularis Oris.

**Figure 7:**
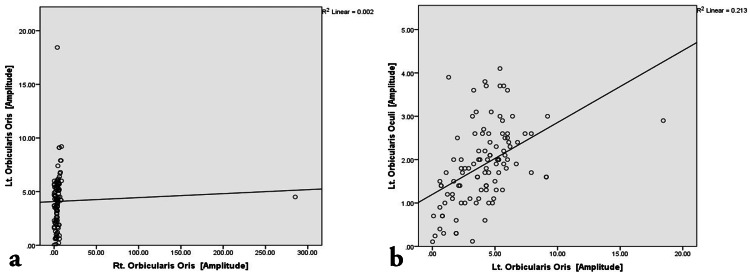
Scatter plot representing the bivariate correlation among affected Orbicularis Oris muscle groups on both sides. (a) Correlation between Right Orbicularis Oris and Left Orbicularis Oris. (b) Correlation between Left Orbicularis Oris and Left Orbicularis Oculi.

The correlation analysis (Figures 4-7) was done to establish the strength of the relationships between variables that result from the common cause. Pearson correlation coefficient value, “r” ranges from -1 to 1 and measures the linear trend between two variables (Table 5). The r =0 implies no linear relationship, r=1 is a perfect positive relationship, and r = −1 is a perfect negative relationship. The positive linear trend is seen if 0 < r < 1 and with samples scattered around the best fit line. The samples will be scattered around the variables if −1 < r < 0.

 

 

**Table 5:** Effect of correlation analysis among the nasalis, orbicularis oris, and orbicularis oculi muscles on both sides

	Rt. Nasalis vs Rt. Orbicularis Oris	Rt. Nasalis vs Rt. Orbicularis Oculi	Rt. Nasalis vs Lt. Nasalis	Rt. Orbicularis Oculi vs Rt. Orbicularis Oris	Rt. Orbicularis Oculi vs Lt. Orbicularis Oculi	Rt. Orbicularis Oris vs Lt. Orbicularis Oris	Lt. Nasalis vs Lt. Orbicularis Oculi	Lt. Nasalis vs Rt. Orbicularis Oris	Lt. Orbicularis Oculi vs Lt. Orbicularis Oris	Lt. Nasalis vs Lt. Orbicularis Oris
Pearson’s Correlation coefficient (r)	-0.082	0.687	0.385	-0.103	0.278	0.041	0.558	0.056	0.461	0.430
P value	0.408	0.0001	0.0001	0.29	0.0001	0.68	0.0001	0.57	0.0001	0.0001
Significance	NS	Highly Significant	Highly Significant	NS	Highly Significant	NS	Highly Significant	NS	Highly Significant	Highly Significant
Strength of correlation	*	Moderately strong positive	Weak positive	*	Very Weak positive	**	Moderately strong positive		Weak positive	Weak positive

The bivariate correlation between the right nasalis and right orbicularis oris was non-significant (weak r-value of -0.082). The bivariate correlation between right orbicularis oculi and right orbicularis oris was non-significant (r-value of -0.103). The bivariate correlation between left orbicularis oris and right orbicularis oris was non-significant (very weak r-value of 0.041). The bivariate correlation between left nasalis and right orbicularis oris was non-significant (r-value of -0.056).

The bivariate correlation between the right nasalis and right orbicularis oculi showed a highly significant moderately strong positive correlation with an r-value of 0.687. The bivariate correlation between the right nasalis and left nasalis showed a highly significant weak positive correlation with an r-value of 0.385. The bivariate correlation between left and right orbicularis oculi showed a highly significant very weak but positive correlation with an r-value of 0.278. The bivariate correlation between left orbicularis oculi and left nasalis showed a highly significant moderately strong positive correlation with an r-value of 0.558. The bivariate correlation between left orbicularis oculi and left orbicularis oris showed a highly significant weak positive correlation with an r-value of 0.461. The bivariate correlation between left nasalis and left orbicularis oris showed a highly significant weak positive correlation with an r-value of 0.430.

## Discussion

The seventh cranial nerve - the facial nerve arises from the brain stem at the cerebellopontine angle and exits the skull through the stylomastoid foramen. It is derived from the second branchial branch during embryogenesis and comprises motor, sensory and parasympathetic nuclei. The associated nuclei are general somatic efferent (GSE), general visceral efferent (GVE), special visceral afferent (SVA), and general somatic afferent (GSA). The GSE innervates the muscles of facial expression. The extratemporal component of the facial nerve terminates into five branches namely the temporal, zygomatic, buccal, mandibular, and cervical branches. Any injury to the facial nerve can range from neurapraxia, axonotmesis, or neurotmesis resulting in a spectrum of disabilities. The damage to the Lower motor neuron leads to facial paralysis which is commonly called BP.

The NCS is an electrodiagnostic test that quantifies the CMAP generated during nerve conduction. In this test, the supramaximal stimuli are applied in front of the ear lobe near the stylomastoid foramen across a standard voltage and the evoked muscle potential is measured as amplitude with representation in units of millivolts. The strength of the amplitude is proportional to the number of muscle fibers present and hence the degenerated motor nerve fiber reflects a reduced amplitude when compared with the normal side.

NCS study is advised to be performed after three days but within 14 days of onset [3,12]. This measurement of delayed recording after 72 hours is advocated as it is the time required for Wallerian degeneration to propagate from the injured intratemporal portion to the distal of the stylomastoid foramen where electrical stimulation is given [13]. Interestingly, though age influences BP treatment outcome when compared to younger patients [14], the duration from onset to recovery is the same for all age groups [15], signifying that no modification in the recording procedure is required and the NCS study is valid across all ages. 

Facial NCSs are used to quantify the degree of nerve damage during the acute phase of nerve injury and as a device to measure the prognosis of recovery by nerve regeneration. ENoG is also useful in counseling the affected individuals on the expected duration of facial weakness and the possible presence of palsy residua [16]. The CMAP measured in millivolts is generated and recorded at the site distal to the stylomastoid foramen by applying supramaximal electrical stimulation on the facial nerve.

In our study, the 104 cases reported were of a broader age range from three to 81 years of age. The CMAP of the affected side was compared to the normal side of the patient and quantified as an amplitude drop percentage. Among age groups, a majority of 35.6% reported cases were under the age of 30 years and the least reported age group was above 60 years with 9.6% cases. The amplitude drop of lesser than or equal to 30% was seen in the majority of 40.38% cases which are mild conduction blocks that could be managed with pharmacotherapy alone. Severe conduction block of greater than or equal to 90% amplitude drop was seen in 23.07% of cases that require surgical nerve decompression along with pharmacotherapy for clinical improvement.

 The relationship between affected muscle groups on both sides was correlated. There was a strong positive correlation between right nasalis versus right orbicularis oculi, and between left nasalis versus left orbicularis oculi muscles. The explanation lies in the anatomy of the facial nerve where the orbicularis oculi are innervated by temporal and zygomatic branches and the Nasalis muscle is innervated by zygomatic and buccal branches [17,18]. The diameter and length of the nerve branch do influence the rate of degeneration, the diameter of the temporal branch is 0.94 mm (± 0.3282) with a length of 30.1 mm (± 6.8995), and a zygomatic branch diameter is 1.002 mm (± 0.4598) with a length of 38.03 mm (± 6.6427). The buccal branch has a diameter and length of 0.99 mm (± 0.3962) and 37.88 mm (± 7.3333), respectively [19]. Therefore, the temporal branch is expected to get degenerated faster due to a shorter diameter and length when compared with other branches. However, in our study, there was a statistically significant association between amplitude drop and the affected side of the face across all age groups. The right orbicularis oris followed by the left orbicularis oris showed higher amplitude loss among muscles. This indicates that axonal loss (AL) is higher in the buccal branch of the facial nerve than in other branches. Also, orbicularis oris is innervated by the mandibular branch of the facial nerve which has to be studied in detail in future studies. Our result was different from the findings of Kwon et al. [7] where the temporal branch was affected more by the resultant increased amplitude drop.

 Nerve conduction results are influenced by factors like the remnant population of intact nerve fibers, the degree of synchronization of muscle fibers, the velocity of stimulus propagation, the velocity of transmission at the neuromuscular junction, the velocity of nerve conduction and impedance between skin and applied electrodes. When ENoG response is not elicited, we should consider other factors like detached electrodes or malfunctioning or total degeneration of the facial nerves before arriving at a diagnostic decision [20]. Hence the final treatment outcome is dictated by the proportion of nerve injury.

 The minimum critical value that suggests an unfavorable prognosis is 90% of CMAP amplitude drop [21] because above which incomplete recovery and a high possibility of secondary synkinesis development in the damaged nerve can happen due to unfavorable regeneration [22]. The CMAP amplitudes of 80% or less have been shown to have complete recovery in six months after treatment but the values of 90% or more had a low recovery rate of 20% only [23]. Sometimes, the rate of degeneration after 90% leads to major sequelae or absence of recovery [24]. The proposal of Fisch [25] that a 90% CMAP amplitude drop within 14 days of palsy onset as a cutoff for decompression of facial nerve through surgical procedures was confirmed by Gantz et al. [26]. Therefore, surgical decompression should be directly performed instead of prolonged pharmacotherapy to improve functional outcomes in cases with 90% degeneration cases as there are no voluntary motor unit potentials. This reduces the delay to initiate treatment and finally improves the quality of life.

Limitations

 In contrast, ENoG cannot differentiate the spectrum of types of nerve damage and hence is useful only in unilateral palsy cases as a modality of a comparison study. The disadvantage of NCSs is that it is expensive and also require skilled technicians to operate this equipment-standardized procedure by controlling dependent variables like skin resistance, placement of electrodes, and applying pressure. This has resulted in a lack of device availability and skilled operating personnel in all hospital settings in developing countries. Considering the advantages, we recommend the NCS as a mandatory procedure before delving into therapeutic modalities in BP patients for proper healthcare delivery.

 Future research needs to be done on other muscles innervated by the facial nerve to understand the extent of degenerative changes during palsy. Additionally, no NCS studies had been conducted in muscles innervated by the cervical branch of the facial nerve till now though neck pain is also a documented clinical feature. Further research has to be directed toward analyzing nerve patency in the later stages of degeneration with proposed follow-up schedules for monitoring the treatment prognosis.

## Conclusions

The NCS is a reliable tool to quantify the degeneration of nerve fibers and also to indicate palsy prognosis when performed after 72 hours of onset. This is useful in the early detection of patients with poor prognostic indicators and thereby formulating treatment plans beyond conventional pharmacotherapy for the improved well-being of the patient. However, a standard guideline with a definitive timeline for later stages of NCS measurement should be determined in future studies.
